# 两个遗传性蛋白C缺陷症家系的分子致病机制研究

**DOI:** 10.3760/cma.j.cn121090-20240628-00236

**Published:** 2025-03

**Authors:** 梦珍 温, 一凡 芦, 媚娜 刘, 朗译 秦, 艳慧 金, 明山 王, 丽红 杨

**Affiliations:** 温州医科大学附属第一医院医学检验中心，浙江省检验诊断及转化研究重点实验室，温州 325015 Department of Laboratory Medicine, the First Affiliated Hospital of Wenzhou Medical University, Key Laboratory of Clinical Laboratory Diagnosis and Translational Research of Zhejiang Province, Wenzhou 325015, China

**Keywords:** 蛋白C, 基因突变, 体外表达, 血栓形成, Protein C, Genetic mutation, In vitro expression, Thrombosis

## Abstract

**目的:**

探究两个遗传性蛋白C（PC）缺陷症家系的分子致病机制。

**方法:**

两位先证者均因静脉血栓栓塞症（VTE）分别于2021年6月和2022年10月就诊于温州医科大学附属第一医院，收集先证者和家系成员临床资料及血样本，检测血浆蛋白C活性（PC∶A）和抗原（PC∶Ag）含量及其他相关凝血指标。以凝血酶生成试验（TGT）评估其抗凝能力。采用DNA直接测序法确定PROC基因突变位点。用生物信息学软件分析突变基因的保守性和致病性。用PyMOL软件分析蛋白质三维模型以及突变氨基酸之间的相互作用。构建野生型和两个突变型表达载体，并瞬时转染HEK293T细胞，提取阳性转染细胞内的细胞总RNA进行突变PROC基因转录水平的研究。采用ELISA法和Western blot和细胞免疫荧光实验进行PC突变蛋白翻译水平的研究。

**结果:**

家系1、2先证者的PC∶A分别降至35％、40％，PC∶Ag分别降至44％、39％，D-二聚体分别升高至4.42 mg/L、0.83 mg/L，其他相关凝血指标无明显异常。测序分析显示，家系1、2先证者的PROC基因第9外显子分别存在c.833T>C（p.Leu278Pro）、c.1330T>C（p.Trp444Arg）杂合错义突变。凝血酶生成试验表明两位先证者及其携带以上PROC基因突变家系成员的抗凝功能均受损。保守性分析显示Leu2278和Trp444在同源物种间呈高度保守。致病性分析显示p.Leu278Pro和p.Trp444Arg均为有害突变。蛋白质模型分析显示该两种突变均能导致蛋白质结构发生改变。体外表达实验显示，与野生型相比，p.Leu278Pro和p.Trp444Arg两种突变在mRNA表达层面没有明显差异，但两种突变导致细胞培养上清液PC∶Ag含量和PC蛋白表达量明显低于野生型，而在细胞培养裂解液中高于野生型。

**结论:**

PROC基因第9外显子杂合错义突变p.Leu278Pro和p.Trp444Arg导致PC蛋白分泌障碍可能是遗传性PC缺陷症的分子致病机制。

## Abstract (en)

**Objective:**

To investigate the molecular pathogenic mechanism of venous thrombosis caused by heterozygous missense mutations in two protein C（PROC）genes through laboratory phenotype analysis, genetic mutation analysis, and in vitro expression experiments.

**Methods:**

Two probands presented with venous thromboembolism at the First Affiliated Hospital of Wenzhou Medical University. Clinical data and blood samples were collected from the probands and their family members to evaluate the plasma protein C（PC）activity（PC∶A）, PC antigen（PC∶Ag）levels, and other relevant coagulation parameters. The anticoagulant capacity was assessed using the thrombin generation test（TGT）. The mutation sites of the PROC gene were identified using direct DNA sequencing. Bioinformatics software was used to analyze the conservation and pathogenicity of the mutated gene. PyMOL software was used for the analysis of the protein three-dimensional models and interactions between mutated amino acids. Wild-type and two mutant expression vectors were constructed and HEK293T cells were transiently transfected. Total cellular RNA was extracted from positively transfected cells to investigate the transcriptional levels of the mutant PROC gene. Enzyme-linked immunosorbent assay, Western blot, and cellular immunofluorescence assays were used to investigate the translation levels of the mutant PROC protein.

**Results:**

Probands 1 and 2 exhibited PC∶A levels of 35％ and 40％ and PC∶Ag levels of 44％ and 39％, with increasing D-dimer levels to 4.42 mg/L and 0.83 mg/L, respectively. Meanwhile, other coagulation parameters revealed no significant abnormalities. TGT demonstrated impaired anticoagulant function in both proband witnesses and their familial PC carriers. Sequencing analysis revealed heterozygous missense mutations c.833T>C（p. Leu278Pro）in proband 1 and c.1330T>C（p. Trp444Arg）in proband 2 within exon 9 of the PROC gene. Conservation analysis revealed that Leu278 and Trp444 were highly conserved across homologous species. Pathogenicity analysis indicated that both p. Leu278Pro and p. Trp444Arg mutations are deleterious. Protein modeling analysis demonstrated that both mutations induce structural alterations in the protein. In vitro expression experiments revealed that compared with the wild-type, both p. Leu278Pro and p. Trp444Arg mutations showed no significant differences in the mRNA expression level of the PC protein. However, both mutations caused significantly lower PC∶Ag content and protein expression levels in the cell culture supernatant compared with the wild-type, whereas higher levels were observed in the cell culture lysate. This indicates the association of both mutations with the secretion function of the PC protein.

**Conclusion:**

The heterozygous missense mutations p. Leu278Pro and p. Trp444Arg in exon 9 of the PROC gene in both probands are associated with decreased PC levels.

蛋白C（PC）是一种由肝脏分泌的依赖维生素K单链糖蛋白，是机体PC抗凝系统的中心成分[1]。当凝血酶与内皮表面的血栓调节蛋白结合时，它会切割重链的Arg12与Leu13之间的激活肽，释放出12个氨基酸残基的小肽，暴露其活性中心，从而PC被激活为活化蛋白C（APC），再由蛋白S（PS）、钙离子和磷脂存在的条件下，APC主要通过灭活活化凝血因子Ⅴ和活化凝血因子Ⅷ来减少凝血酶的产生发挥其抗凝、促纤溶和抗炎的作用[2]–[3]。遗传性PC缺陷症是由位于常染色体2q13-q14的PROC基因缺陷引起的常染色体显性或不完全显性遗传性疾病[4]。遗传性PC缺陷症患者静脉血栓栓塞症（VTE）的风险增高[5]，VTE主要包括下肢深静脉血栓形成（DVT）和肺栓塞，后者是除冠心病和中风之外导致心血管疾病死亡的第三大原因[6]。遗传性PC缺陷症在静脉血栓患者中约占8％[7]。本研究报告两个遗传性PC缺陷症家系并探讨其分子致病机制。

## 对象和方法

1. 研究对象：本研究纳入2021年6月和2022年10月在温州医科大学附属第一医院就诊的两例遗传性蛋白C缺陷症患者及其家系。其现病史和既往史。选择同期100名健康体检者作为对照组（女45名、男55名，年龄22～56岁），建立本研究实验室凝血指标的生物参考值。本研究通过本院伦理委员会的审查（KY2022-R193）并取得所有受试者知情同意。

2. 标本采集与处理：两个家系所有患者均在抗凝治疗前采集其外周静脉血2.7 ml，用109 mmol/L枸橼酸钠以1∶9抗凝处理，2 000×*g*离心15 min，取上层乏血小板血浆用于检测凝血指标和凝血酶生成试验；下层血细胞用于提取DNA，进行PCR扩增。

3. 血浆凝血指标检测：通过Stago STA-R Max全自动血凝仪采用发色底物法检测先证者及其家系成员的PC活性（PC∶A）和抗凝血酶活性（AT∶A），免疫比浊法检测D-二聚体（D-D），凝固法检测PS活性（PS∶A），试剂为Stago原装配套试剂盒。采用ELISA法检测PC抗原（PC∶Ag）含量，试剂盒购自温州长风生物技术有限公司。

4.凝血酶生成实验：使用Stago自动校正凝血酶曲线法（CAT）检测凝血酶生成，试剂为Stago配套的FluoCa试剂盒，20 µl ppp试剂，用1 pmol/L组织因子启动凝血，绘制60 min内正常血浆和受试者血浆的凝血酶生成曲线，推导其内源性凝血酶生成潜力。

5. PCR扩增：用酚-氯仿法提取先证者及其家系成员基因组DNA，PCR扩增仪为ABI Thermalcycler 2720。所用引物序列参照文献[6]，PCR反应体系为25 µl。提取试剂盒购自北京天根生化科技有限公司（TIANamp Genomic DNA Kit），按照说明书进行操作。

6. PROC基因测序：PCR扩增产物送上海桑尼生物科技有限公司测序。用Chromas软件将测序结果与GenBank中的PROC序列进行比对，对可疑突变进行反向测序验证。明确突变位点后，检测家系其他成员相应的外显子片段。

7. 生物信息学分析：通过多序列比对软件ClustalX-2.1-win将突变氨基酸与另外几种同源物种的氨基酸序列进行比对，分析突变氨基酸在同源物种中的保守性（同源物种氨基酸序列来源：http://www.ncbi.nim.nih.gov/homplogene）。用Mutation Taster、Polyphen-2、PROVEAN及FATHMM在线生物信息学软件预测突变位点对PC结构的影响。采用PyMOL软件构建突变前后的蛋白结构模型，分析突变对蛋白质空间结构的改变以及影响，PC的晶体结构数据来自蛋白质数据库（http://www.rcsb.org/pdb/home.do, PDB ID:1AUT）。

8. 构建突变表达载体、细胞培养和转染：PC空载和野生型（PC-WT）质粒均购自南京擎科生物科技有限公司，选用TK-PCDH-copGFP-T2A-Puro构建PC表达质粒，采用定点诱变试剂盒（美国STRATAGENE公司产品）在PC-WT的基础上构建p.Leu278Pro突变型表达质粒（PC-L278P）和p.Trp444Arg突变型表达质粒（PC-W444R），对整个PC的cDNA进行全长测序，发现定点突变成功引入，且没有引入其他突变。HEK293T细胞解冻复苏后转移至含10％胎牛血清的DMEM培养基，于37 °C、5％二氧化碳环境中常规培养。利用转染试剂盒LipofectamineTM 3000 reagent（美国Thermo Fisher公司产品）将空载、野生型和突变型表达载体瞬时转染生长状态良好的HEK293T细胞，在荧光显微镜下观察转染效率。

9. PROC基因转录水平检测：细胞转染48 h后采用Trizol试剂盒提取细胞裂解液中的总RNA，采用Prime Script逆转录试剂盒，将RNA逆转录成cDNA，根据TB Green® Premix Ex Taq™ II FAST qPCR试剂说明书通过实时荧光定量PCR（qRT-PCR）法检测野生型和突变型PC mRNA的表达水平。PC扩增引物序列：正向5′-CCAGCTCCTCTTGACTCAGTG-3′，反向5′-AGGAAGGAGTTGGCACGTTT-3′。内参基因GAPDH扩增引物序列：正向5′-AGAAGGCTGGGGCTCATTTG-3′，反向5′-AGGGGCCATCCACAGTCTTC-3′。用Bio-Rad CFX Manager 3.1软件分析PROC基因和GAPDH的Ct值，采用比较Ct法（2^-ΔΔCt^）计算野生型和突变型PROC基因的mRNA相对表达量。

10. PROC基因蛋白表达水平检测：收集转染阳性细胞，用含有蛋白酶降解抑制剂的RIPA缓冲液裂解细胞，将变性后的细胞培养裂解液和上清液中的蛋白通过10％的SDS聚丙烯酰胺凝胶电泳分离转移至醋酸纤维膜上，通过封闭以及一抗和二抗孵育后显影检测。采用ELISA试剂盒（购自上海江莱科技有限公司）检测细胞培养上清液和细胞裂解液中野生型和突变型的PC∶Ag水平。

11. 细胞免疫荧光：转染48 h后的细胞经PBS清洗后，依次用4％的多聚甲醛（上海碧云天公司产品）固定20 min，加入0.1％ Triton X-100（上海碧云天公司产品）室温孵育20 min渗透破膜，之后加入1∶200稀释的兔抗人PC IgG单克隆抗体（美国Thermo Fisher公司产品）在4 °C条件下孵育12 h；采用1∶500稀释的Cy3标记的山羊抗兔抗体（美国Proteintech公司产品）在37 °C条件下孵育2 h。最后用一定量含DAPI的抗荧光淬灭剂（上海碧云天公司产品）进行封片。使用Thunder荧光显微镜成像系统（德国Leica公司产品）进行细胞免疫荧光成像。

## 结果

1. 临床资料：家系1先证者，男，55岁，货车司机，因左下肢发紫伴疼痛两周至我院血管外科就诊，以“左下肢DVT”收住院。凝血检查示D-D 4.42 mg/L（参考值0～0.5 mg/L），PC∶A、PC∶Ag分别降至35％、44％，其弟弟、女儿、侄子的PC∶A和PC∶Ag均明显降低，其他相关凝血指标均正常。肝、肾功能正常。该家系共3代9人纳入本研究。经家系调查发现先证者父亲在50岁时出现脑静脉窦血栓，经抢救无效死亡。其余家庭成员无血栓栓塞史。家系图见图1。家系成员凝血指标检测结果见表1。

**图1:**
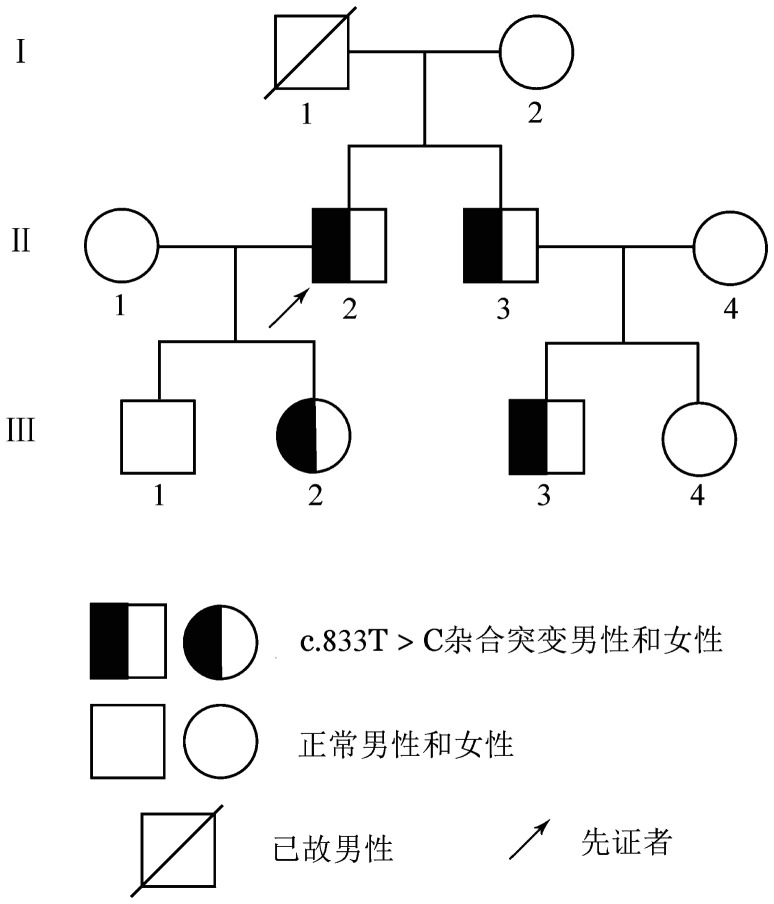
遗传性蛋白C缺陷症家系图（家系1）

**表1:** 遗传性蛋白C缺陷症家系1凝血指标检测结果

家庭成员	年龄（岁）	D-D（mg/L）	PS∶A（％）	AT∶A（％）	PC∶A（％）	PC∶Ag（％）
母亲（Ⅰ2）	79	0.41	78	98	94	104
先证者（Ⅱ2）	55	4.42	88	100	35	44
妻子（Ⅱ1）	52	0.24	87	103	111	105
弟弟（Ⅱ3）	51	0.58	96	99	47	51
弟媳（Ⅱ4）	50	0.31	91	113	107	110
儿子（Ⅲ1）	29	0.14	101	115	104	106
女儿（Ⅲ2）	25	0.46	92	102	46	52
侄子（Ⅲ3）	26	0.39	95	110	41	49
侄女（Ⅲ4）	22	0.31	88	107	108	105

参考值		0～0.5	70～130	98～118	70～130	70～135

**注** D-D：D-二聚体；PS∶A：蛋白S活性；AT∶A：抗凝血酶活性；PC∶A：蛋白C活性；PC∶Ag：蛋白C抗原

家系2先证者，男，63岁，有吸烟史40余年，每天约20支，高血压4年，因摔倒后卧床1周出现心悸气促至我院急诊就诊，肺动脉造影显示：右肺动脉，左下肺动脉及部分分支多发栓塞。双下肢动静脉B超示：左下肢深静脉栓塞。凝血指标检查：D-D轻度升高（0.83 mg/L），PC∶A、PC∶Ag分别降至40％、39％，其女儿和外孙PC∶A和PC∶Ag也有不同程度降低，其他相关凝血指标均正常，该家系3代7人纳入研究。该家系其他成员均否认血栓栓塞病史。该家系图见图2，凝血指标检测结果见表2。

**表2:** 遗传性蛋白C缺陷症家系2凝血指标检测结果

家庭成员	年龄（岁）	D-D（mg/L）	PS∶A（％）	AT∶A（％）	PC∶A（％）	PC∶Ag（％）
先证者（Ⅱ1）	63	0.83	92	98	40	39
妻子（Ⅱ2）	61	0.24	93	103	98	102
儿子（Ⅲ1）	33	0.24	89	114	94	97
女儿（Ⅲ2）	29	0.25	91	99	49	50
女婿（Ⅲ3）	32	0.31	85	105	95	101
外孙（Ⅳ1）	4	0.26	90	108	43	42
外孙女（Ⅳ2）	6	0.28	87	113	99	109

参考值		0～0.5	70～130	98～118	70～130	70～135

**注** D-D：D-二聚体；PS∶A：蛋白S活性；AT∶A：抗凝血酶活性；PC∶A：蛋白C活性；PC∶Ag：蛋白C抗原

**图2:**
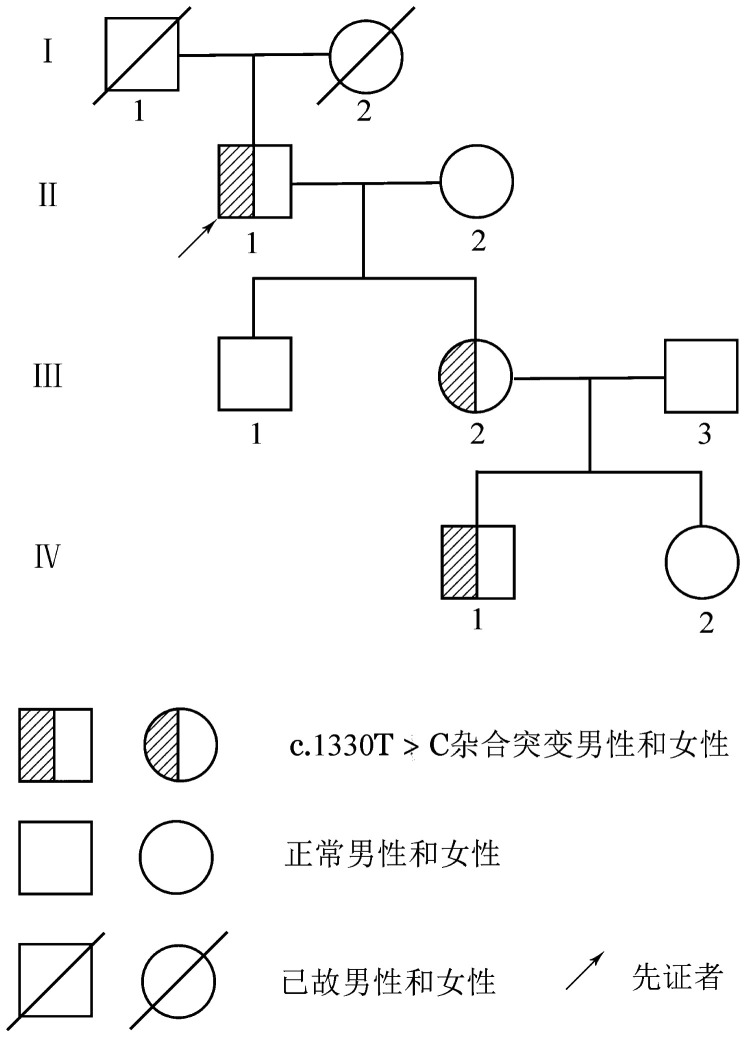
遗传性蛋白C缺陷症家系图（家系2）

2. 凝血酶生成实验结果：与正常对照相比，两例先证者及其家系中PROC基因突变携带者的内源性凝血酶电位和峰高均升高，其中两位先证者升高较明显（图3）。

**图3:**
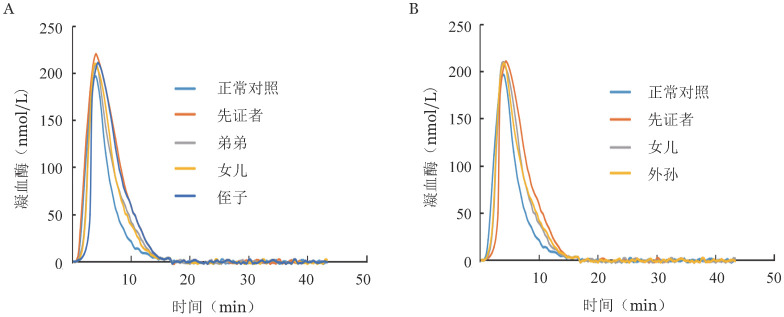
遗传性蛋白C缺陷症家系1（A）和家系2（B）凝血酶生成曲线

3.基因组DNA测序结果：家系1先证者及其弟弟、女儿和侄子的PROC基因第9号外显子均存在c.833T>C杂合错义突变（导致p.Leu278Pro），该突变2021年由本课题组首次报道[8]。家系2先证者及其女儿、外孙PROC基因第9号外显子均存在c.1330T>C杂合错义突变（导致p.Trp444Arg）。两个家系其他受检成员均为野生型。详见图4。在千人基因组计划（https://www.genome.gov/27528684/1000-genomes-projec）中未发现p.Trp444Arg，排除基因多态性的可能；查阅人类基因突变数据库HGMD（https://www.hgmd.cf.ac.uk/ac/all.php）和国内外相关文献，未见p.Trp444Arg报道。

**图4:**
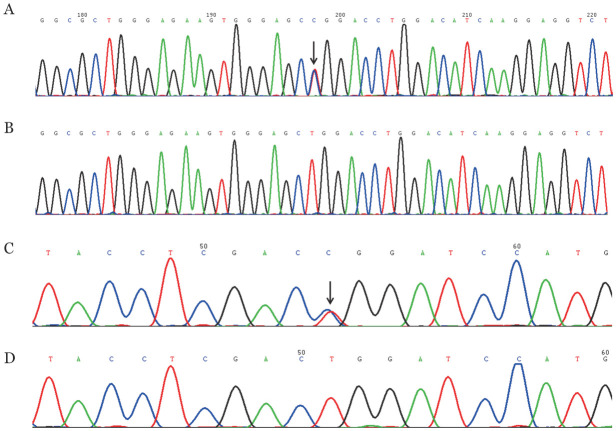
遗传性蛋白C缺陷症家系PROC基因第9号外显子测序结果 **A** c.833T>C杂合错义突变；**B** c.833T>C野生型；**C** c.1330T>C杂合错义突变；**D** c.1330T>C野生型

4. 生物信息学分析结果：保守性分析显示Trp444位点与其他9种同源物呈高度保守（图5）。Mutation Taster、PROVEAN、Polyphen-2和FATHMM在线生物信息学软件分析结果均提示p.Trp444Arg突变为有害突变。蛋白模型显示p.Trp444Arg突变导致苯环结构消失、侧链变长（图6）。

**图5:**
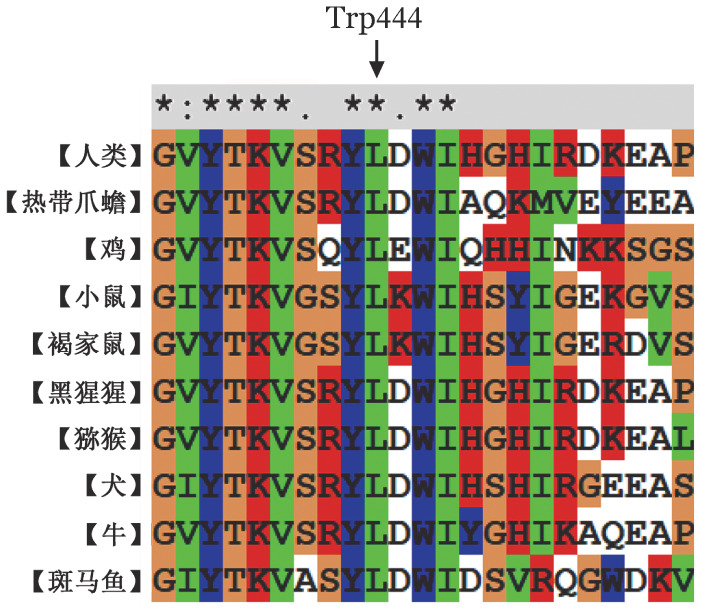
p.Trp444Arg PROC基因序列的进化保守性分析

**图6:**
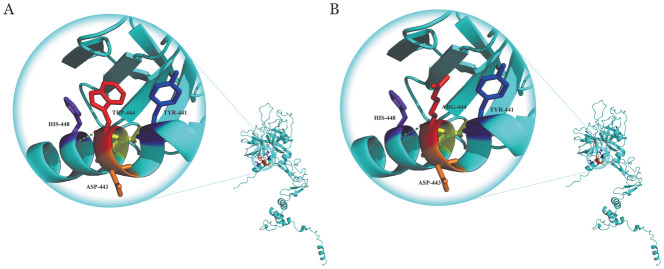
蛋白C蛋白模型分析图 **A** p.Trp444Arg野生型；**B** p.Trp444Arg突变型 **注** 绿色虚线为氢键

5. 体外表达实验结果：转染野生型和突变型表达载体48 h后在同一视野下观察荧光显微镜下普通光镜视野和荧光视野结果如图7所示，野生型和突变型表达载体均转染成功，且转染效率基本一致，约为85％。qRT-PCR结果显示，转染48 h后的HEK293T细胞与PC-WT相比，PC-L278P和PC-W444R的mRNA表达水平差异无统计学意义（*P*值分别为0.938、0.182），表明p.Leu278Pro和p.Trp444Arg两个位点的突变均不影响PROC基因转录（图8）。ELISA法检测转染后HEK293T细胞裂解液和上清的PC∶Ag表达量（图9），以PC-WT表达载体转染的HEK293T细胞的细胞培养裂解液和上清液中的PC∶Ag含量为100％，PC-L278P表达载体细胞培养裂解液和上清液中的PC∶Ag含量分别为（118.1±1.9）％、（33.7±3.3）％（*P*＝0.003），PC-W444R表达载体细胞培养裂解液和上清液PC∶Ag含量分别为（132.3±1.4）％、（5.7±0.45）％。Western blot结果显示，PC-L278P和PC-W444R表达载体的细胞裂解液中的含量明显高于野生型，而在细胞上清液中PC-L278P表达量明显低于野生型，PC-W444R表达果量几乎为0（图10）。利用Cy3对转染质粒的HEK293T细胞中的PC蛋白进行染色。然后用免疫荧光显微镜观察细胞内是否存在残留/异常的PC蛋白，结果显示PC-WT蛋白均分布在细胞质中，PC-L278P和PC-W444R蛋白均匀分布在细胞质核中，少部分分泌到细胞质中，但PC-W444R分泌至胞质的量较少，在相同参数下荧光信号明显较弱（图11）。

**图7:**
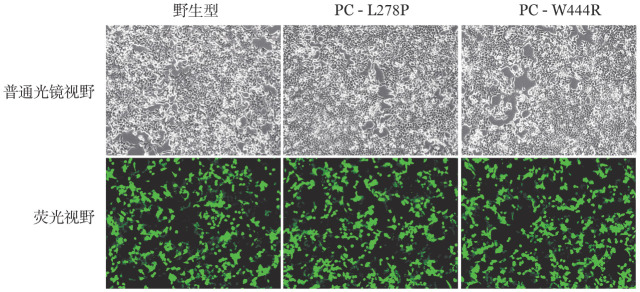
细胞转染图

**图8:**
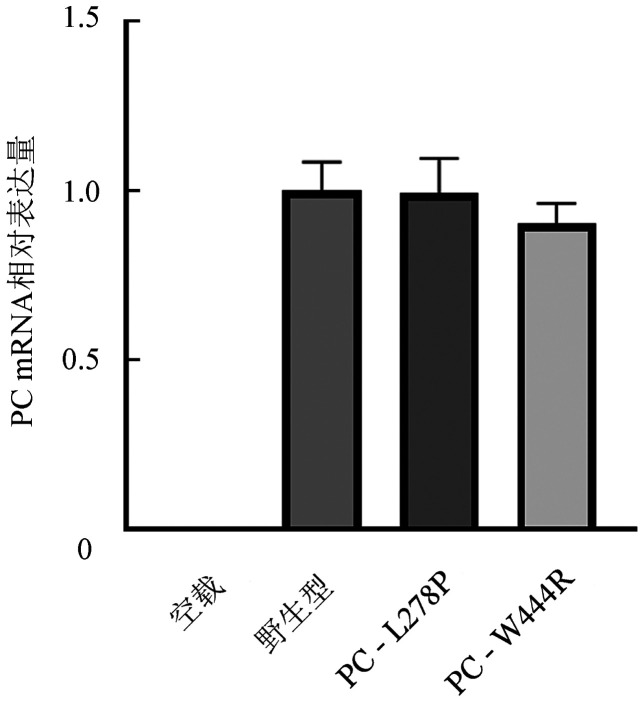
转染细胞内蛋白C（PC）的mRNA表达水平

**图9:**
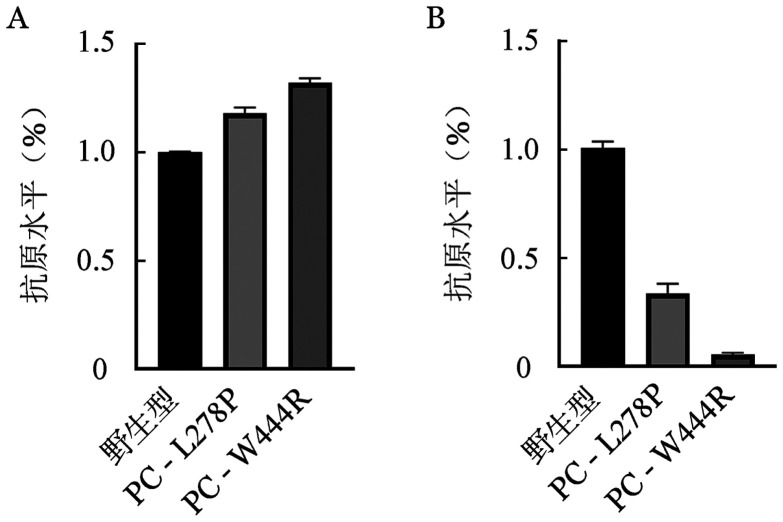
重组蛋白的ELISA结果 **A** 细胞裂解液蛋白C抗原水平；**B** 细胞上清液蛋白C抗原水平

**图10:**
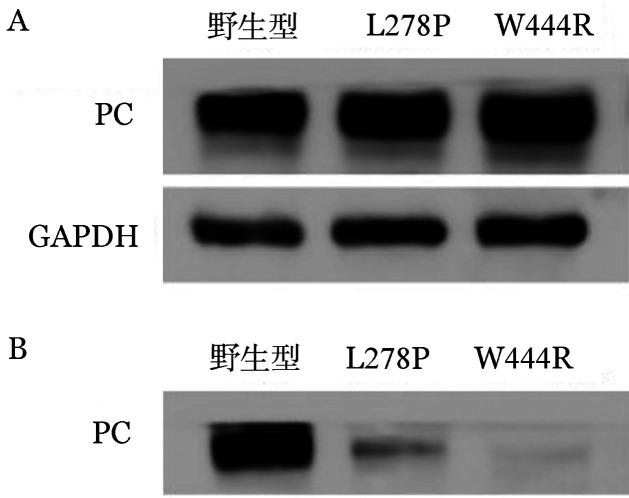
p.Leu278Pro和p.Trp444Arg突变型蛋白免疫印迹分析结果（A细胞裂解液；B培养上清液）

**图11:**
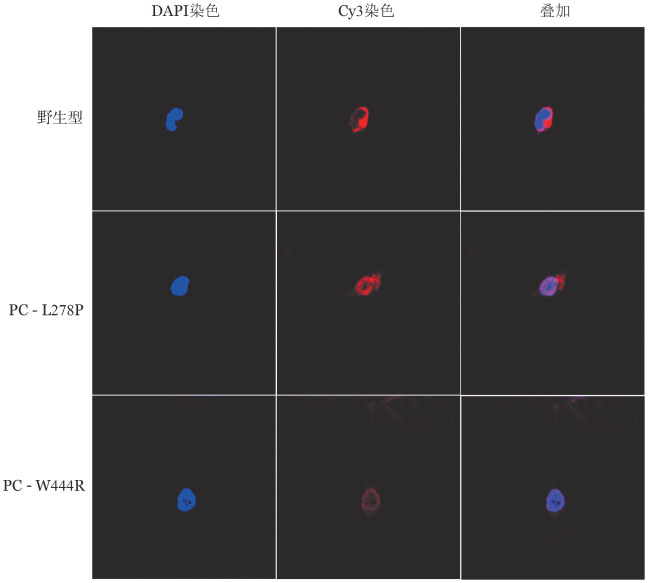
重组蛋白的细胞免疫荧光分析结果（细胞核用蓝色荧光显示，PC蛋白用红色荧光显示）

## 讨论

VTE是由获得性因素（肥胖、制动、高龄、手术、妊娠、吸烟等）和遗传性易栓症共同作用造成[9]。亚洲人的VTE主要与PC、PS和抗凝血酶（AT）缺陷有关[10]。PC缺陷症的诊断主要依靠实验室对PC∶A和PC∶Ag检测。根据PC∶A水平和PC∶Ag降低程度的不同，可将遗传性PC缺陷症分为Ⅰ型和Ⅱ型：Ⅰ型为PC分子合成减少，表现为PC∶A水平和PC: Ag含量同步降低；Ⅱ型为合成异常的分子，表现为PC∶A水平降低，但PC∶Ag含量正常[11]。在本研究中，家系1先证者及弟弟、女儿、侄子PC∶A水平和PC∶Ag含量同步降低，家系2先证者及其女儿、外孙PC∶A水平和PC∶Ag含量同步降低，2个家系均属于Ⅰ型遗传性PC缺陷症。凝血酶生成试验表明两例先证者及其家系中PROC基因突变携带者的抗凝血活性均受损，其中两位先证者表现更明显。通过基因测序发现家系1先证者及其弟弟、女儿、侄子PROC基因第9外显子均存在p.Leu278Pro杂合错义突变，家系2先证者及其女儿、孙子第九外显子存在p.Trp444Arg杂合错义突变，排除肝肾疾病和其他导致PC∶A水平和PC∶Ag含量降低的因素，以上两种杂合错义突变可能分别与两个家系PC水平降低有关。

成熟PC蛋白由419个氨基酸组成，由一条21kD轻链与一条41kD重链经Cys141和Cys277之间的二硫键连接而成[12]。PC轻链N末端的γ-羧基谷氨酸结构域是与Ca^2+^结合的区域，为凝血酶-血栓调节蛋白复合物激活PC的必需区域；重链C末端的胰蛋白酶样丝氨酸蛋白酶结构域为PC的活性区域[13]–[14]。本研究的p.Leu278Pro和p.Trp444Arg均位于此活性区域。p.Leu278Pro由Xu等[8]首次报道，其PC蛋白模型分析显示，p.Leu278Pro突变导致Pro278和Leu265之氢键增加，形成新的苯环。同时，Huang等[15]等通过对多种蛋白结构研究发现，带有芳香基团的氨基酸也可能作为蛋白质折叠反应的抑制剂。新的苯环形成可能会使得蛋白质的折叠速率下降，从而影响蛋白空间结构；他们还发现蛋白质的折叠动力学也与残基的疏水特性显著相关，随着疏水氨基酸残基数量的增加，蛋白质折叠速率降低，因此，对于p.Trp444Arg而言，当带正电的极性氨基酸Arg取代非极性氨基酸Trp后，苯环结构消失，侧链变长，也可能会影响二级结构的稳定以及蛋白质的折叠。PC的第212～461位的氨基酸组成了其催化结构域，APC底物在切割之前必须与位于其中的底物结合区结合[16]。因此，位于此区域的p.Leu278Pro和p.Trp444Arg两个突变导致的空间结构改变可能也改变了PC的催化结构域稳定性，从而影响PC的催化效率及底物与蛋白酶的结合。

本研究构建了PC-L278P和PC-W444R表达载体瞬时转染HEK293T细胞，结果表明两个突变均不影响PROC基因的转录。为研究这两个突变对PC蛋白表达水平的影响，对两个突变载体转染后的HEK293T细胞裂解液和上清液进行Western blot试验分析，结果显示两个突变型PC蛋白在裂解液中的含量均高于野生型，而在上清夜中的含量均低于野生型，表明p.Leu278Pro和p.Trp444Arg两种突变均能影响PC蛋白由细胞内向细胞外的分泌。同时，ELISA和细胞免疫荧光结果也显示两个PC突变蛋白在细胞内含量较高，与Western Blot结果一致。

遗传性PC缺陷症大多是由于PROC单一位点的杂合突变所致，纯合和复合杂合突变较为罕见，纯合子患者的PC活性非常低或无法检测到（通常小于1％）[17]，临床症状一般出现在出生后2 h到几个月，伴有危及生命的血栓并累及中枢神经系统、眼睛、肾脏和皮肤（暴发性紫癜）[18]。本研究两位先证者携带PROC基因突变杂合子，PC水平约为正常人的50％，家系1先证者下肢DVT可能与p.Leu278Pro杂合错义突变以及长期制动有关。家系2先证者发生严重DVT和PE，可能与p.Trp444Arg杂合错义突变、长期吸烟、制动及高龄等易栓因素的共同作用有关。住院期间，家系1先证者连续3 d皮下注射那屈肝素钙抗凝治疗，之后改为口服利伐沙班15 mg每日2次，连续20 d，复查双下肢血管B超显示双下肢深静脉血栓较前好转，出院后利伐沙班改为20 mg/d，三个月后改为10 mg/d。家系2先证者接受连续8 d皮下注射低分子肝素钙抗凝治疗后，影像学显示肺血栓栓塞无明显改善，遂改为口服利伐沙班15 mg每日2次，3周后复查肺动脉造影示血栓较前好转，利伐沙班剂量调整为20 mg/d，未再发生血栓事件。

综上所述，PROC基因第9外显子杂合错义突变p.Leu278Pro和p.Trp444Arg导致PC蛋白分泌障碍可能是本研究两个遗传性PC缺陷症家系的分子致病机制。
